# Characterization of an *Escherichia coli*-derived triple-type chimeric vaccine against human papillomavirus types 39, 68 and 70

**DOI:** 10.1038/s41541-022-00557-y

**Published:** 2022-10-31

**Authors:** Ciying Qian, Yurou Yang, Qin Xu, Zhiping Wang, Jie Chen, Xin Chi, Miao Yu, Fei Gao, Yujie Xu, Yihan Lu, Hui Sun, Jingjia Shen, Daning Wang, Lizhi Zhou, Tingting Li, Yingbin Wang, Qingbing Zheng, Hai Yu, Jun Zhang, Ying Gu, Ningshao Xia, Shaowei Li

**Affiliations:** 1 State Key Laboratory of Molecular Vaccinology and Molecular Diagnostics, School of Life Sciences, School of Public Health, Xiamen University, Xiamen, 361102 China; 2 National Institute of Diagnostics and Vaccine Development in Infectious Diseases, Xiamen University, Xiamen, 361102 China

**Keywords:** Immunology, Protein vaccines

## Abstract

In vaccinology, a potent immunogen has two prerequisite attributes—antigenicity and immunogenicity. We have rational designed a triple-type HPV vaccine against HPV58, −33 and −52 covered in Gardasil 9 based on the sequence homology and similar surface loop structure of L1 protein, which is related to cross-type antigenicity. Here, we design another triple-type vaccine against non-vaccine types HPV39, −68 and −70 by immunogenicity optimization considering type specific immunodominant epitopes located in separate region for different types. First, we optimized the expression of wild-type HPV39, −68 and −70 L1-only virus-like particles (VLPs) in *E. coli* through N-terminal truncation of HPV L1 proteins and non-fusion soluble expression. Second, based on genetic relationships and an L1 homologous loop-swapping rationale, we constructed several triple-type chimeric VLPs for HPV39, −68 and −70, and obtained the lead candidate named H39–68FG-70DE by the immunogenicity optimization using reactivity profile of a panel type-specific monoclonal antibodies. Through comprehensive characterization using various biochemical, VLP-based analyses and immune assays, we show that H39–68FG-70DE assumes similar particulate properties as that of its parental VLPs, along with comparable neutralization immunogenicity for all three HPV types. Overall, this study shows the promise and translatability of an HPV39/68/70 triple-type vaccine, and the possibility of expanding the type-coverage of current HPV vaccines. Our study further expanded the essential criteria on the rational design of a cross-type vaccine, i.e. separate sites with inter-type similar sequence and structure as well as type-specific immunodominant epitope to be clustered together.

## Introduction

Cervical cancer has become the fourth-most prevalent cancer in women and poses a serious threat to women’s health^1–3^. Indeed, according to the World Health Organization (WHO), in 2020, ~ 604,127 women were newly diagnosed with cervical cancer worldwide, with ~341,831 deaths. Studies have shown that the main cause of cervical cancer and other genital cancers is persistent infection with high-risk human papillomavirus (HR-HPV)^4–6^. To date, over 450 distinct HPV types have been identified and classified into 4 groups by the WHO International Agency for Research on Cancer (IARC) working group according to their ability to generate malignancies^7–9^: class 1 (carcinogenic) includes HPV16, −18, −31, −33, −35, −39, −45, −51, −52, −56, −58 and −59; class 2 A (probably carcinogenic) includes HPV68; class 2B (possibly carcinogenic) includes HPV26, −30, −34, −53, −66, −67, −69, −70, −73, −82, −85 and −97; class 3 (not classifiable) includes HPV6 and −11, and class 4 is considered probably non-carcinogenic to humans^10,11^. Class 1 and 2 HPV types are highly correlated with cervical cancer and are classified as high-risk (HR-HPV), whereas class 3 types, HPV6 and −11, are mainly associated with genital warts and are classified as low-risk types (LR-HPV)^8,12^.

Five HPV vaccines have been licensed to date: three bivalent vaccines (Cervarix [GlaxoSmithKline], Cecolin [Innovax] and Recombinant Human Papillomavirus Bivalent [Types 16, 18] Vaccine [Pichia pastoris] [Walvax Biotechnology Co., Ltd.]), a quadrivalent vaccine (Gardasil [Merck & Co.]) and a nonavalent vaccine (Gardasil 9 [Merck & Co.]). Of these, Gardasil 9 offers the most protection, and is effective against ~90% of cervical cancer induced by 7 types of HR-HPV (HPV16, −18, −31, −33, −45, −52 and −58) as well as against 90% of genital warts induced by LR-HPV types (HPV6 and −11)^13,14^. Despite the broad spectrum of protection offered by these marketed vaccines, approximately 10% of cervical cancer caused by other HR-HPV types still cannot be effectively prevented^15^. Furthermore, with time and with the increasing popularity of vaccine administration against HPV, the prevalence of the non-vaccine types showed increasing to some extent in several types. In a study, these non-vaccine HPV types (HPV39, −51, −56 and −59) were found to have increased prevailing ratio in some regions where Gardasil were extensively used for ten years^16^. A recent meta-analysis by Mesher et al. also showed an increase in HPV39, −52, −53 and −73 in the post-Gardasil vaccine era, and among those 20–24 years of age, evidence indicated increased prevalence of HPV70^17^. Consequently, there is a demand for vaccines with broader prevention efficacy.

According to the WHO report, the global prevalence of HR-HPV39, −68 and −70 accounted for 1.5%, 0.8% and 0.2% respectively in the cases of cervical cancer in 2020^11^. HPV39 ranks ninth among the ten most frequent HPV oncogenic types in Chinese women with cervical cancer, and in southern parts of China, HPV39 prevails just after HPV52, −16, −58 and −18^18^. In other regions, the prevalence of HPV39 is similar, ranking eighth frequent HPV carcinogenic types in developed countries, and ranking tenth in developing regions. HPV68 and −70 also prevail globally after HPV 39, despite of relatively lower frequency^11^. Given the HPV39, −68 and −70 prevailing while not being protected by Gardasil 9, a next-generation of HPV vaccine should have broader coverage and preferably includes these three carcinogenic HPV types.

As a non-enveloped, double-stranded DNA virus, the HPV capsid is composed of the major structural protein, L1, and the minor structural protein, L2. L1 proteins can self-assemble into virus-like particles (VLPs) and induce a strong immune response^19–21^. The five marketed vaccines are all based on L1-only VLPs^22,23^:Cervarix^24,25^ Cecolin^26,27^, and a HPV16/18 bivalent vaccine produced in Pichia pastoris by Walvax Biotechnology Co., Ltd. contain VLPs for HPV16 and −18; Gardasil comprises VLPs for HPV6, −11, −16 and −18^28,29^, with Gardasil 9 adding in VLPs for HPV31, −33, −45, −52 and −58^14^. Each vaccine is formulated through a mixture of VLP types meaning that they are essentially type-specific and offer limited broad-spectrum protection. One could predict that including additional HPV VLPs into the vaccine production strategy could broaden the protection rate; however, studies show that as the number of VLP types increases, the protein content in the vaccine also increases substantially, which, in turn, requires the administration of a much higher dose of vaccine^30^. Already, one dose of Gardasil 9 contains up to 270 µg of total HPV L1 proteins. For humans, the injection of an excessively high dosage of a foreign protein can induce significant side effects. In addition to dosage considerations, adding more HPV types will increase the complexity of the vaccine production process, and may generate immune competition among the different VLPs^31^. Increasing the number of VLP types is therefore considered a very challenging approach.

Nearly all of the marketed vaccines offer protection by inducing neutralizing antibodies; however, for highly antigenically variable pathogens, such as HPV, HIV and influenza, traditional vaccine strategies provide limited broad-spectrum protection against these variations^32–34^. Recent innovation in structural vaccinology suggest that protein structural information can be used to optimize and achieve precise antigen functional epitopes that can be harnessed for immunogen design. Indeed, studies show that the effective presentation and/or integration of functional epitopes can trick the innate immune spectrum into producing more complex, broad-spectrum responses^35^. One example is relatively conserved immunodominant epitopes were grafted onto viral surface proteins using a germline-targeting strategy in the development of universal HIV vaccines^36^. Another method widely used in vaccine design is computationally optimized broadly reactive antigen method, or COBRA^37^. The principle in this method is to construct a consensus sequence from multiple immunodominant sequences from different strains to achieve cross-type protection^38^. For influenza viruses, a recombinant hemagglutinin (HA) polypeptide constructed by fusing the sequences from H5N1 and H1N1 can be used to elicit a broadly reactive immune response against both H5N1 and H1N1 influenza strains (patent no. EP2616545A4). Another vaccine, Human COBRA 2, designed by incorporating key neutralizing epitopes of two HA antigens-Human COBRA 2 (Hu-CO) and Human-Avian COBRA 2 (Hu-Av CO)-also elicits broadly protective antibodies against heterologous clades of viruses^39^.

Both linear and conformational epitopes have been identified on the surface of HPV L1 VLPs^40^, whereas most of neutralizing antibodies recognize those conformational epitopes that comprise some of the five surface loops^41,42^. For instance, the well-known HPV16.V5 is an HPV16-specific neutralizing antibody, which showed immune predominant frequency in the sera of native HPV-infected humans and is of clinical importance^43^. HPV16.V5 was identified to recognize the FG loop (aa266~297) and HI loop (aa339~365) of L1 protein, and could neutralize virus infection by interfering with the endocytosis of virus entry^44^. The immune complex structure of HPV16.U4, HPV16.14J and HPV16.1A with HPV16 VLP also showed that the neutralizing epitope of HPV L1 was located at the five surface loop regions (BC, DE, EF, FG and HI)^45,46^. Surface loops of genetically close HPV types are known to have a highly similar conformation^47^. In previous studies, we designed a chimeric VLP of the genetically close HPV58/33/52 based on sequence homology of L1 proteins and structural information of the surface loops, which elicited high levels of cross-neutralizing antibodies in both mice and non-human primates^48^. However, why single chimera VLP could work well for producing cross-neutralization against three HPV types, and which loop could be an ideal target for clustering remains to be determined. Here, we demonstrate the potent elicitation of the neutralizing antibody depends not only the resurface epitope success, but also its immunodominant nature for producing antibody, and the potential universality of this design through the characterization of a single-particle cross-type vaccine candidate that can simultaneously prevent carcinogenic types HPV39, −68 and −70. Using type-specific monoclonal antibodies and pseudovirus platforms, we analyzed the antigenicity and immunogenicity of this cross-type candidate vaccine, and confirmed that the HPV loop transplantation strategy is feasible between phylo-genetically close HPV types. Remarkably, genetically close types means the types belong to a same phylogenetic group, whereas genetically distant types fall in different group in the phylogenetical tree of HPV by the alignment of L1 amino acid sequences^48^. Dominant loop transfer provides a new level of antigenicity and immunogenicity for the chimeric VLP without destroying the backbone types, thus achieving cross-type protection with fewer immunogens. This chimeric VLP also fills the present gap in HPV prevention against HPV39/68/70.

## Results

### Sequence identity and prevalence of HPV39, −68 and −70

Using data from the 2021 WHO epidemiological statistics report^11^, Supplementary Fig. 1a depicts the prevalence of 25 potentially carcinogenic HPV types (class 1 and 2) and low-risk HPV6 and −11 among women with invasive cervical cancer in China and across the globe. HPV16 and −18 are most frequently associated with cervical cancer, and are covered by all 5 of the commercially available vaccines. In contrast, the prevalence of HPV35/39/59—not presently covered by any vaccine—is second only to the types covered by Gardasil 9.

We divided the 27 HPV types into 8 phylo-genetically close groups according to the evolutionary distance of the L1 protein. As shown in Supplementary Fig. 1b, HPV39, a class 1 type, which has a close genetic relationship in the Alpha 7 species to HPV68 and HPV70, both of which are class 2 HPV types (probably or possibly carcinogenic) and neither of which is covered in any of the presently available vaccines. In terms the degree of the amino acid sequence identity of the surface loops—an important consideration for chimeric design—we noted that the 5 homologous surface loops of HPV39, −68 and −70 have the same numbers of amino acids (Supplementary Fig. 1c; vertical orientation). Furthermore, of the loops, the BC loop has a difference of only 3 amino acids, whereas loops DE, EF, FG and HI each have 8 amino acid differences. Therefore, we selected HPV39, −68 and −70 for chimeric vaccine design through homologous loop-swapping.

### Expression, purification, and characterization of HPV 39, −68 and −70 wild-type VLPs

**Fig. 1:**
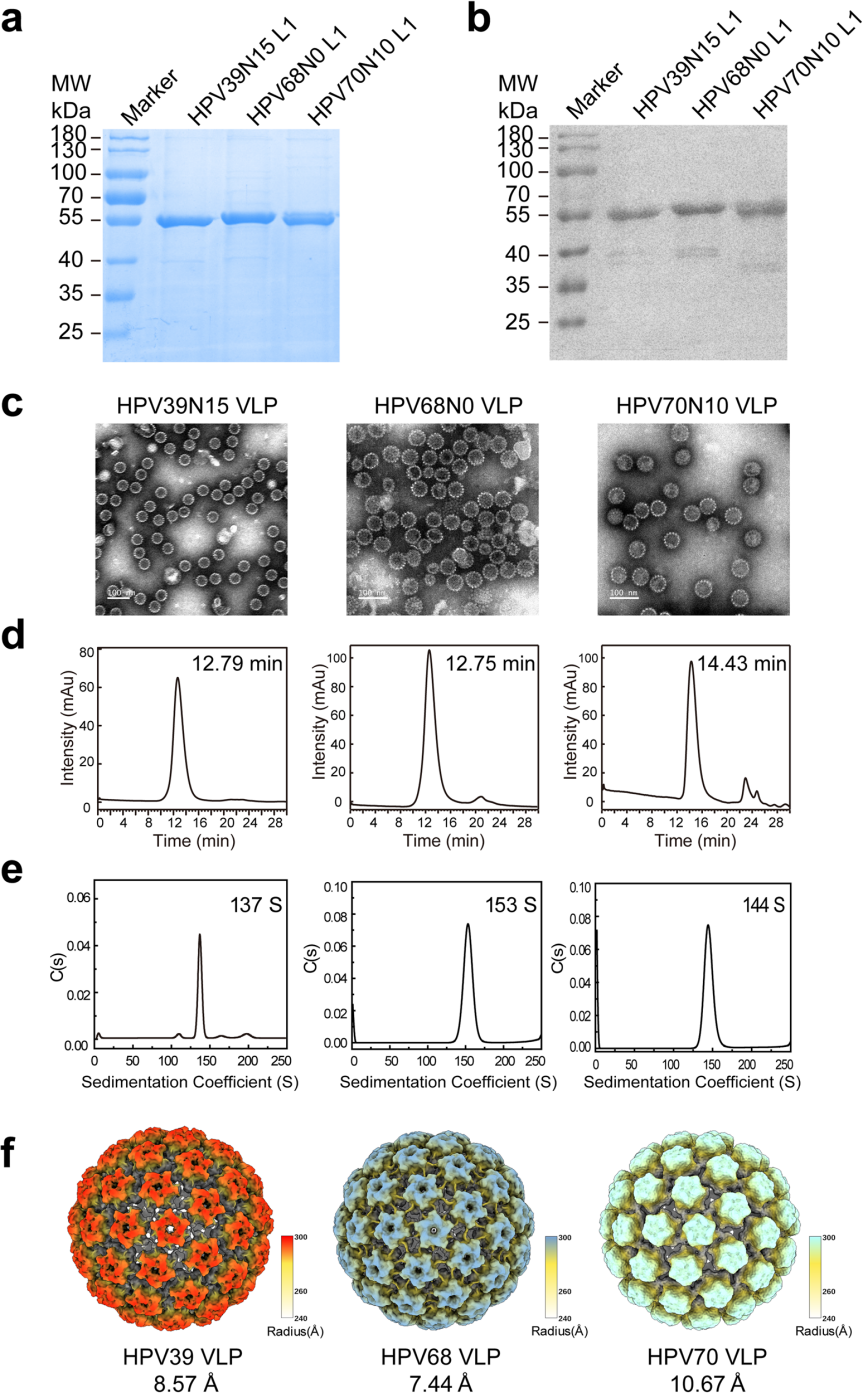
Characterization of the HPV39, −68 and −70 WT VLPs. The WT N-terminally truncated L1 proteins were subjected to reducing SDS-PAGE (**a**) and western blotting (**b**) with a wide-spectrum linear mAb 4B3. Transmission electron microscopy (TEM) images, scale bar, 100 nm (**c**), high-performance size-exclusion chromatography (HPSEC) profiles (**d**), analytical ultracentrifugation sedimentation (AUC) profiles (**e**), and reconstructed cryo-EM maps (**f**) of the WT L1-only VLPs. Cryo-EM structures of HPV39, −68 and −70 VLPs (radially colored from 240 Å to 300 Å) were determined at final resolutions of 8.57 Å, 7.44 Å and 10.67 Å, respectively.

HPV39, −68 and −70 genes for the L1 proteins were cloned into the pTO-T7 plasmid and expressed in the *E. coli* system. Full-length and a series of N-terminal truncations (5-, 10-, 15-aa) of HPV39, −68 and −70 L1 proteins were constructed to optimize expression. Soluble and highly expressed constructs with a purity of >95%—HPV39 (15–505 aa), HPV68 (0–505 aa) and HPV70 (10–506 aa)—were purified by two-step chromatography. The molecular weights were determined to be ~55 kD for all three constructs (Fig. 1a, b). Upon removing the reducing agent (dithiothreitol), the purified L1 proteins underwent self-assembly into VLPs, and the particle properties of the three WT VLPs were characterized and confirmed using transmission electron microscopy (TEM), high-performance size-exclusion chromatography (HPSEC), analytical ultra-centrifugation (AUC), and cryo-electron microscopy (cryo-EM). All three WT VLPs displayed high homogeneity (Fig. 1c). The retention times for all three samples in HPSEC was ~12 min (Fig. 1d), and the sedimentation coefficients for HPV39 (15–505 aa), HPV68 (0–505 aa) and HPV70 (10–506 aa) were 137 S, 153 S and 144 S, respectively (Fig. 1e). The three WT VLPs were subjected to vitrification and cryo-EM analysis. The particles demonstrating high homogeneity were selected for three-dimensional (3D) reconstruction using AUTO3DEM^49^ or cisTEM^50^ software. Finally, the icosahedral density maps of the HPV39, HPV68 and HPV70 capsids at resolutions of 8.57 Å, 7.44 Å and 10.67 Å, respectively, were obtained (Supplementary Fig. 2 and Supplementary Table 1). As shown in Fig. 1f, all three WT VLPs, which show similar structural features to those reported previously for HPV16 and HPV18^26^, present as a typical T = 7 icosahedral capsid shell consisted of 72 pentamers and with a diameter of 60 nm.

**Table 1:** Half-effective dosage (ED_50_) of double-type and triple-type chimeras in mice.

Antigen category	HPV VLP	ED_50_ (μg)		
		anti-HPV39	anti-HPV68	anti-HPV70
Wild-type control	HPV39	0.022	>0.900	–
	HPV68	>0.900	0.023	–
	HPV39&68	0.021	0.023	–
Double-type chimera	H39–68FG	0.091	0.405	–
	H68-39BC	0.395	0.021	–
	H68-39HI	>0.900	0.028	–
Wild-type control	HPV39	0.019	>0.900	>0.900
	HPV68	>0.900	0.021	0.900
	HPV70	>0.900	>0.900	0.019
	HPV39&68&70	0.021	0.021	0.021
Triple-type chimera	H39–68FG-70DE	0.021	0.030	0.040
	H39–68FG-70EF	0.900	0.024	0.611
	H39–68FG-70HI	0.022	0.148	0.047

### Construction and characterization of HPV39/68 chimeric VLPs

**Fig. 2:**
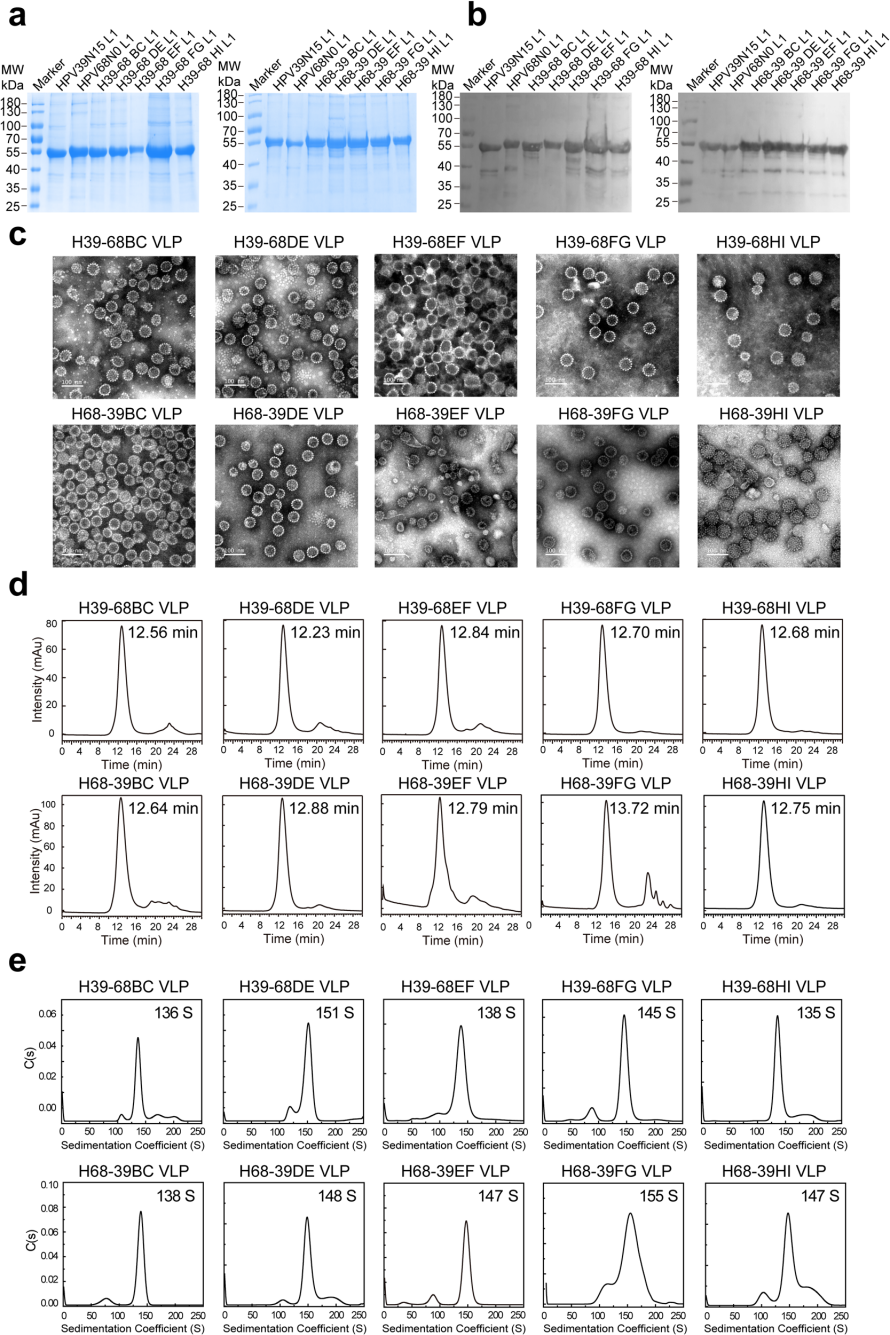
Characterization of H39–68 and H68-39 chimeric VLPs. WT and chimeric L1 proteins were subjected to reducing SDS-PAGE (**a**) and western blotting (**b**) with a wide-spectrum linear mAb 4B3. **c** Transmission electron microscopy (TEM) images of HPV39/68 chimeric VLPs. Scale bar, 100 nm. High-performance size-exclusion chromatography (HPSEC) profiles (**d**) and analytical ultracentrifugation sedimentation (AUC) profiles (**e**) of the chimeric VLPs.

Despite the loop structures on the pentamer surfaces display significant conformational differences among various HPV types, the overall core regions of L1 pentamers are almost identical, and the surface loops are more conserved among genotypes within the same species group rather than between groups, which offers utility for homologous loop swapping^47,51^. We prioritized the replacement of homologous loops one at a time between HPV39 and HPV68, because these genes have a closer genetic relationship among the three HPV types^52^. To this end, we constructed 10 chimeras: H39–68BC, H39–68DE, H39–68EF, H39–68FG, H39–68HI, H68-39BC, H68-39DE, H68-39EF, H68-39FG and H68-39HI, and expressed these chimeras in *E. coli*. L1 proteins with 95% purity were then purified by two-step column chromatography (Fig. 2a, b) and allowed to self-assemble in vitro. Through TEM (Fig. 2c), HPSEC (Fig. 2d) and AUC (Fig. 2e), we found that the double-type chimeric VLPs have good homogeneity similar to the WT VLPs.

**Fig. 3:**
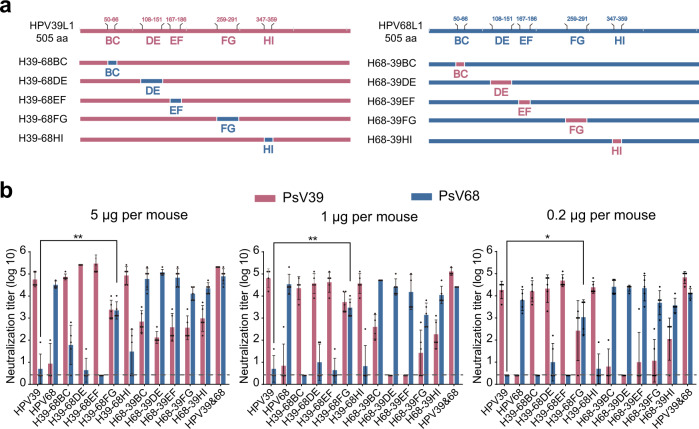
Immunogenicity of H39–68 and H68-39 chimeric VLPs. **a** Schematic representation of the chimeric HPV39/68 L1 proteins based on WT HPV39 and HPV68 L1. **b** Immunogenicity of HPV39/68 chimeric VLPs in BALB/c mice delivered at three dosages. BALB/c mice (*n* = 5) were inoculated intraperitoneally with a high (5 μg/dose), middle (1 μg/dose), or low (0.2 μg/dose) dose of WT HPV39, HPV68 and HPV39/68 chimeric VLPs at weeks 0, 2, and 4. Neutralization antibody levels at week 8 after the first immunization were tested by neutralization assays. The dotted line indicates the limit of detection for the assay. All data were analyzed by one-way analysis of variance (ANOVA) and are presented as the mean ± standard deviation (SD); *P < 0.05, **P < 0.01; ***P < 0.001; ****P < 0.0001. The error bars reflect the SD and symbols represents individual mice.

We then further assessed the immunogenicity of these chimeric HPV39/68 VLPs. BALB/c mice were inoculated by intraperitoneal injection three times with chimeric VLPs formulated with aluminum adjuvant. The neutralization titers of the sera after three rounds of immunization were measured with a pseudovirus-based neutralization assay (PBNA). We found a dose-dependent immunization effect for both WT VLPs and HPV39/68 chimeric VLPs (Fig. 3a, b), with higher titers found in the high-dose group as compared with the low-dose group. Several HPV39/68 chimeras could elicit neutralizing antibodies (nAbs) against the types corresponding to the heterologous loops without destroying the ability of the backbone type to elicit homologous nAbs. In contrast, very limited cross-nAb titers (2.5–400) could be elicited between WT HPV39 and WT HPV68, indicating obvious type specificity. Notably, the titers of heterologous nAbs elicited by chimeric VLPs with different heterologous surface loops were different and dose-dependent: anti-HPV68 titers elicited by H39–68FG ranged from 160 to 5120 (0.2 μg group, *P* < 0.05), 1280 to 10,240 (1 μg group, *P* < 0.01) and 1280 to 10240 (5 μg group, *P* < 0.01); these were all significantly higher than those elicited by the WT HPV39 VLPs (anti-HPV68 titers of 2.5 to 80). Similarly, H68-39BC and H68-39HI elicited high neutralizing titers against HPV39 (titers ranging from 2.5 to 3200, 2.5 to 5120, respectively) as compared with WT HPV68 VLPs (anti-HPV39 titers of 2.5–400).

H39–68FG, H68-39BC and H68-39HI all exhibited good double-type cross-neutralization potency. Therefore, we next subjected these chimeras to further study to determine the half-effective dose (ED_50_); i.e., the minimum effective dose at which 50% of the experimental animals seroconverted in a given group^53^. As shown in Table 1 and Supplementary Table 2, the ED_50_ values for WT HPV39 and HPV68 were 0.022 μg and 0.023 μg, respectively. The chimeric VLPs similarly provided neutralization activity; albeit, with differing levels. For H39–68FG, the ED_50_ value for HPV39 was 0.091 μg; for H68-39BC and H68-39HI, the ED_50_ values for HPV68 were 0.021 μg and 0.028 μg, respectively; these results indicate that these chimeras offered neutralization activity against the backbone type of the chimera to levels similar to that of the WT HPV39 or HPV68. In contrast, H39–68FG provided lower ED_50_ against HPV68 (ED_50_ = 0.405 μg) than HPV39 against HPV 68 (>0.900 μg), and H68-39BC provided lower ED_50_ against HPV39 (ED_50_ = 0.395 μg) than HPV68 against HPV 39(>0.900 μg), suggesting that a single surface loop grafted onto a heterologous backbone type also has the ability to induce neutralizing antibodies against the donor loop type. Thus, the chimeric VLPs, H39–68FG and H68-39BC, with “one anti-two” cross-neutralization activity, could be used as vaccine antigens for the generation of vaccines.

### Construction and characterization of triple-type HPV39/68/70 chimeric VLPs

**Fig. 4:**
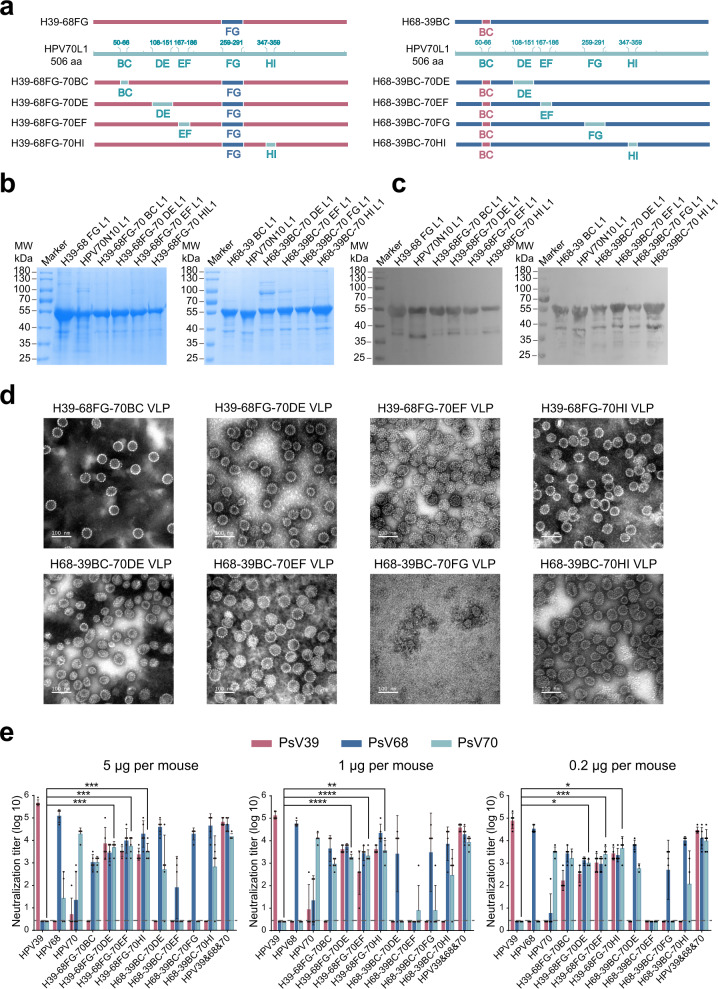
Characterization of HPV39/68/70 chimeric VLPs. **a** Schematic representation of the chimeric HPV39/68/70 L1 proteins based on H39–68FG or H68-39BC L1. The WT and chimeric L1 proteins were subjected to reducing SDS-PAGE (**b**) and western blotting (**c**) with a wide-spectrum linear mAb 4B3. **d** Transmission electron microscopy (TEM) images of HPV39/68/70 chimeric VLPs. Scale bar, 100 nm. **e** Immunogenicity of HPV39/68/70 chimeric VLPs in BALB/c mice. BALB/c mice (n = 5) were inoculated intraperitoneally with high (5 μg/dose), middle (1 μg/dose) or low (0.2 μg/dose) doses of WT HPV39, −68, −70, or HPV39/68/70 chimeric VLPs at week 0, 2, and 4, and neutralization antibody levels at week 8 after the first immunization were tested by neutralization assays. The dotted line indicates the limit of detection for the assay. All data were analyzed by one-way analysis of variance (ANOVA) and are presented as the mean ± standard deviation (SD), *P < 0.05, **P < 0.01; ***P < 0.001; ****P < 0.0001. The error bars reflect the SD and the symbols represents the individual mice.

To further verify that homologous loop swapping can endow the backbone VLP with the ability to elicit nAbs against the heterologous loop type, we engineered HPV70 into the H39–68FG and H68-39BC double-type chimeras, as HPV70 is the closest homolog (Supplementary Fig. 1b). We therefore individually swapped each of the remaining four loops with that from HPV70. This loop swapping produced 8 triple-type chimeras: H39–68FG-70BC, H39–68FG-70DE, H39–68FG-70EF, H39–68FG-70HI, H68-39BC-70DE, H68-39BC-70EF, H68-39BC-70FG and H68-39BC-70HI (Fig. 4a). We found that these chimeras each displayed high purity, good particle morphology, and homogeneity (Fig. 4b–d and Supplementary Fig. 3).

We next sought to test the cross-neutralization efficacy of these triple-type chimeras. Groups of female BALB/c mice at 6 weeks of age were immunized on weeks 0, 2, and 4 with the triple-type chimeric VLPs or WT HPV39, −68 and −70 VLPs formulated with aluminum adjuvant. Among the eight chimeras, the four with H68-39BC as the backbone failed to elicit cross-neutralization against HPV70 and, in some cases, also showed a loss of neutralization activity against HPV39. The remaining 4 chimeras with H39–68FG as the backbone were able to trigger cross-neutralization against all three HPV types. In particular, H39–68FG-70DE, H39–68FG-70EF and H39–68FG-70HI were able to elicit high cross-nAb titers against HPV70 in the high-dose group, with titers of 10^3.7^, 10^3.7^ and 10^3.5^, respectively (*P* < 0.001), compared with WT HPV39 (Fig. 4e). These levels were slightly decreased in the middle-dose and low-dose groups but were still significantly higher as compared with WT HPV39.

We then went on to measure the ED_50_ values for the three triple-type chimeras to test their immunogenicity against infection. We found that all three chimeras could induce seroconversion in half of the mice against HPV39, -68 and -70 infections at very low dosages (Table 1 and Supplementary Table 3). H39–68FG-70DE, in particular, provided cross-neutralization at a level equivalent to that of the combined administration of WT HPV39, −68 and −70, with ED_50_ values of 0.021 μg (anti-HPV39), 0.030 μg (anti-HPV68) and 0.040 μg (anti-HPV70). H39–68FG-70EF and H39–68FG-70HI also showed good triple-type cross-neutralization and could prevent HPV39, −68 and −70 infections. Overall, H39–68FG-70DE and H39–68FG-70HI were chosen as the best cross-type vaccine candidates for further study.

### Antigenicity and immunogenicity of HPV39/68/70 cross-type vaccine candidates

**Fig. 5:**
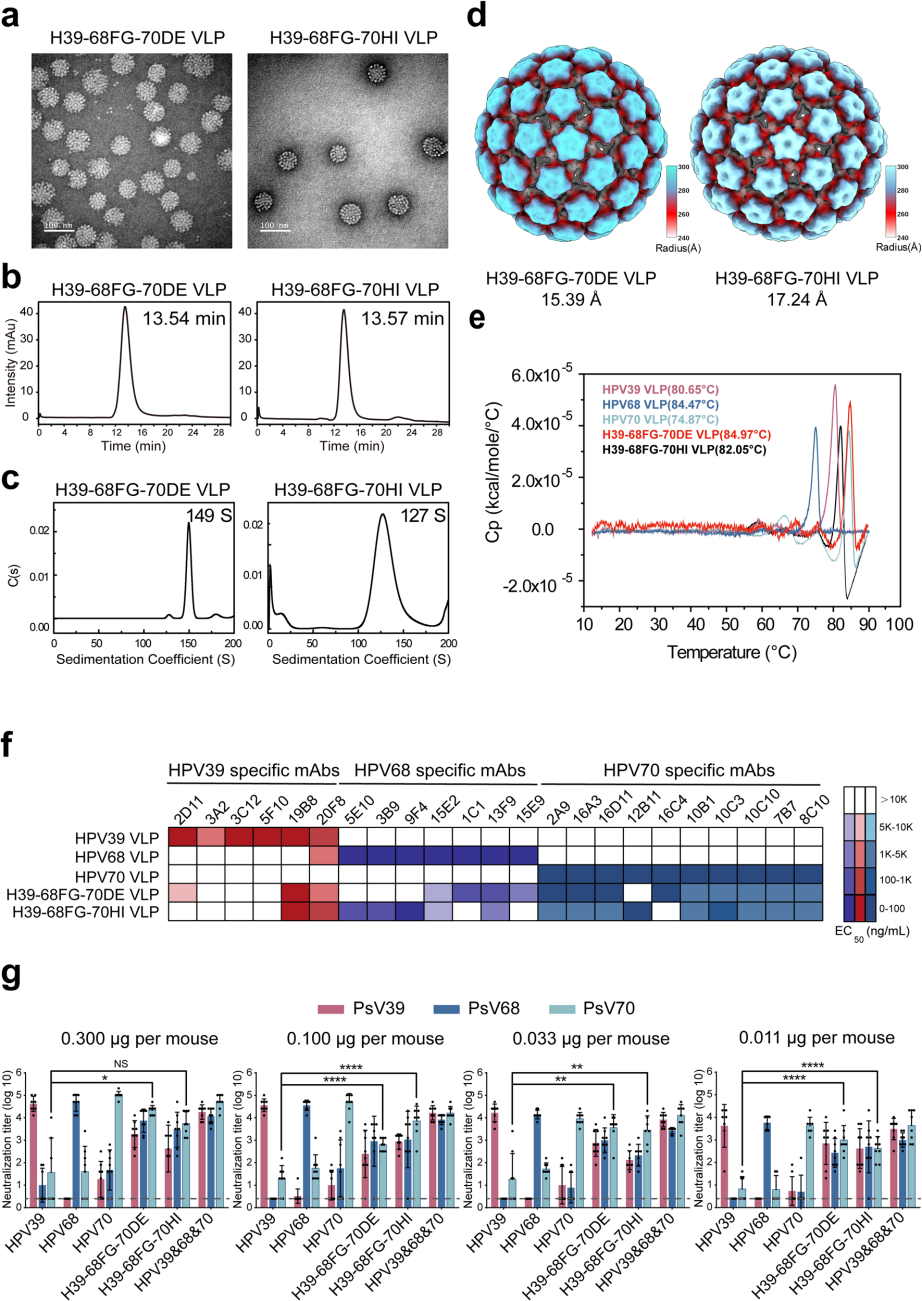
The properties, antigenicity, and immunogenicity characterization of triple-type chimeric single-particle vaccine candidate molecules. Transmission electron microscopy (TEM) images, scale bar, 100 nm (**a**), high-performance size-exclusion chromatography (HPSEC) profiles (**b**), analytical ultracentrifugation sedimentation (AUC) profiles (**c**), differential scanning calorimetry (DSC) profiles (**d**) and cryo-EM structures (radially colored from 240 Å to 300 Å) of H39–68FG-70DE and H39–68FG-70HI chimeric VLPs (**e**). **f** Heatmap representations of the EC_50_ values of HPV39/68/70 chimeric VLPs based on ELISA assays against a type-specific mAb panel of HPV39, −68 and −70 VLPs. The key indicates the heatmap gradient. A detailed characterization of each mAb is shown in Supplementary Table 4. **g** Immunogenicity of HPV39/68/70 cross-type vaccine candidate in BALB/c mice. BALB/c mice (*n* = 8) were inoculated intraperitoneally with one of four doses (0.300, 0.100, 0.033 or 0.011 μg/dose) of HPV39/68/70 cross-type vaccine candidate at weeks 0, 4, and 8. The neutralization antibody levels at week 10 after the first immunization were tested by neutralization assays. The dotted line indicates the limit of detection for the assay. All data were analyzed by one-way analysis of variance (ANOVA) and are presented as mean ± standard deviation (SD); *P < 0.05, **P < 0.01; ***P < 0.001; ****P < 0.0001. The error bars reflect the SD and the symbols represent the individual mice.

We next sought to assess batch consistency, and confirmed that both of the cross-type vaccine candidates, H39–68FG-70DE and H39–68FG-70HI, were produced with excellent quality and consistency of purity, particle morphology, and homogeneity among batches (Fig. 5a–c). We also determined the cryo-EM structures of the chimeric VLPs H39–68FG-70DE and H39–68FG-70HI at resolutions of 15.39 Å and 17.24 Å, respectively (Supplementary Fig. 2 and Supplementary Table 1). As shown in Fig. 5d, the chimeric VLPs present as a T = 7 icosahedral structure similar to the WT VLPs as expected. Finally, we measured the melting temperatures (Tm) of the chimeric and WT VLPs using differential scanning calorimetry (DSC). As shown in Fig. 5e, the thermal stabilities from strongest to weakest were H39–68FG-70DE, HPV68, H39–68FG-70HI, HPV39 and HPV70, indicating that the chimeric VLPs had similar or even better thermal stability than the WT VLPs.

To investigate the antigenicity of H39–68FG-70DE and H39–68FG-70HI, we selected a range of type-specific monoclonal antibodies (mAbs) against WT HPV39/68/70 VLPs. This mAb panel was then used to determine the median effective concentration (EC_50_) of binding via ELISA (Supplementary Table 4). We observed the binding of HPV39 mAbs against the chimeras diminished to varied extent (Fig. 5f), suggesting that the recognition sites for these mAbs was located at or near the loops. Notably, binding of H39–68FG-70DE and H39–68FG-70HI to HPV39-specific mAbs (3A2, 3C12, 5F10 [both], 2D11 [70HI only]) was lost, indicating roles for DE, FG and HI loops of HPV39 may involve some neutralization epitopes that are recognized by these HPV39-specific mAbs. Instead, HPV39 chimeras bearing both of a single loop of HPV68 (FG) and HPV70 (DE/HI) could rescue the binding activities of HPV68 and HPV70-specific mAbs that could not react with WT HPV39 VLPs. And H39–68FG-70DE shows overall higher reactivity with HPV70 specific mAbs than H39–68FG-70HI. In sum, the translocation of specific surface loops can translocate antibody binding reactivity.

Finally, we characterized the immunogenicity of the two chimeric VLPs in mice at lower dosages (0.300, 0.100, 0.033, 0.011 μg). As shown in Fig. 5g, the two chimeric VLPs maintained stable triple-type cross-neutralization activity, even at the lowest dosage of 0.011 μg. nAb titers against HPV39/68/70 were recorded as 10^2^-10^3^, confirming that these cross-type chimeric vaccines offer advantages over traditional type-specific vaccines in terms of cross-neutralization. Overall, we find H39–68FG-70DE to be the best triple-type chimeric VLP, consistent to its overall better reactivity profile against the HPV70 mAb panel. and suggest its potential utility as a vaccine antigen candidate for the prevention of HPV39/68/70 infection.

## Discussion

Rational immunogen design is expected to solve the current challenges facing vaccine development^54^. As well-known in vaccinology, there are two prerequisite attributes for a potent immunogen, i.e. antigenicity based on specific epitope structure and immunogenicity raised from those immunodominant epitopes. The effectiveness of most vaccines depends on nAb response; however, most protective nAbs are type-specific. Therefore, for pathogens that frequently mutate or with multiple genotypes, such as HPV, it is becoming increasingly important to determine new ways to optimize neutralizing epitopes while achieving broad immune protection. At present, there are several immunogen design strategies in use for the development of broad-spectrum vaccines, such as germline targeting, COBRA, consensus sequence construction, and structural biology-based epitope transplantation. For instance, structural analysis of multiple variants of the factor H binding protein (fHbp) on the surface of *Neisseria meningitidis* (MenB) led to the identification of key functional epitopes on different variants, and the successful design of a new epitope through transplantation, offering broad protection against group B meningitis infection^55,56^. These methods could be explored in the development of HPV vaccines against multiple types of viruses.

In our previous study, we designed a triple-type HPV chimera based on higher sequence homology of three types of L1 proteins and similar structures of the five surface loops. The chimera VLP is derived from a basic type of HPV VLP (HPV58 as example) with two surface loops swapped by HPV33 BC and HPV52 HI, which have similar loop conformation and fewer sequence discrepancy between each other types. We established the proof-of-concept of the relational design of triple-type HPV vaccine. However, we did not address why single chimera VLP could work well for producing cross-neutralization against three HPV types, and which loop could be an ideal target for clustering remains to be determined, which is beyond the structural information, because the potent elicitation of the neutralizing antibody depends not only the resurface epitope success, but also its immunodominant nature for producing antibody. In this study, we showcase another chimera HPV39/68/70 by further optimization in the second attribute, i.e. the immunogenicity related to type specific immunodominant epitopes located in separate region for different types.

Here, we engineered a single VLP immunogen that contained the immunodominant regions of three HPV genotypes, focusing on those genotypes not already covered or presumably only partly covered by existing vaccines. Based on a consensus sequence strategy, we successfully integrated the immunodominant epitopes of HPV types 39, 68 and 70 through epitope transplantation, and elicited protective antibodies against all the three serotypes. We sought to explain the reasons for the successful of this epitope exchange strategy from two aspects of antigenicity and immunogenicity. First, in comparing the immunogenicity of WT and chimeric VLPs, we identified that all five surface loops played a role in eliciting nAbs. Through our analysis, we show that the immunodominant region of each HPV type is distinct, and that alternative immunogenic epitopes of variants 2 or 3 could be grafted onto the backbone of variant 1, offering cross-neutralization. We show that the FG loop is the dominant neutralizing epitope of HPV 68, when we then swapped the FG loop of HPV68 into HPV39, the chimeric H39–68FG elicited a neutralizing response against HPV68, with high antibody titers. Comparatively, we find that the DE and HI loops of HPV70 elicit nAbs against HPV70, and integrated these loops into the double-type chimera. In terms of antigenicity, H39–68FG-70DE shows overall higher reactivity with HPV70 specific mAbs than H39–68FG-70HI (5 of the 10 HPV70-specific antibodies had lower EC_50_ values [<50 ng/mL] with H39–68FG-70DE rather than that of H39–68FG-70HI), the region covering DE loop might be more immunogenic than HI loop in HPV70 L1 and therefore enables the H39–68FG-70DE to elicit higher and balanced triple-type neutralizing antibody response both in ED_50_ and antibody titration assays. Thus, we further underscore the immunological consideration-separate immunodominant site for various types or pathogens should be clustered-on chimera design that is usually based on sequence and structural information. Overall, we show that by simply translocating just 16 amino acids, we could successfully construct a triple-type chimeric VLP that can induce cross-neutralization against HPV 39/68/70 at the same time.

In addition to eliciting excellent triple-type cross-neutralization, the chimeric VLPs constructed in this study provide obvious advantages in terms of immune dosage, achieving a level of cross-neutralization at a lower dosage that is comparable with or even better than that of the multivalent vaccine. For instance, with the chimera, 5 μg H39–68FG-70DE is needed to produce nAbs against three constitutive HPV types. Comparatively, using WT VLPs—which can only elicit nAbs against itself—require separate single doses of 5 μg HPV39, 5 μg HPV68 and 5 μg HPV70 VLP, and, thus, a total of 15 μg of foreign protein. In addition, the produce of chimera could dramatically simplify the vaccine production complexity from three WT VLPs to one chimeric VLP, especially under Good Manufacturing Practice of Medical Products (GMP) regulation. On the other hand, the chimera VLP actually cluster major neutralization sites of three HPV types into single VLP and might diminish the non-neutralization antibody elicitation than three WT VLPs as immunogen. Moreover, based on the evolutionary relationship, the progressive chimeric antigen screening strategy development in this study has greatly improved the screening efficiency of candidate vaccines. Theoretically, there are at least 60 triple-type chimeric VLP combinations achievable through surface loop swapping. Yet, using the strategy presented in this study—preferentially screening double-type chimeric VLPs with a closer genetic relationship and then adding in the third—reduced the number of chimeras that needed to be verified. Considering that at least 25 HPV types are highly related to cervical cancer, if the antigen-antibody interaction sites can be precisely located, a wider spectrum of protection can be achieved by transplanting fewer amino acids and fusing more types of immunodominant epitopes.

In summary, we verified the feasibility and effectiveness of homologous loop-swapping as a strategy to induce cross-neutralization among different but closely related HPV types, and obtained a vaccine molecule H39–68FG-70DE that can achieve triple-type cross-neutralization to prevent infection from high-risk carcinogenic HPV39/68/70. The finding and analysis about the chimera of HPV39, −68 and −70 in this study could further expand the knowledge and consideration on HPV chimera vaccine design from the immunological aspect, going beyond the sequence and structural information. Future clinical work will ascertain the feasibility and commercial utility of this H39–68FG-70DE chimera.

## Methods

### Ethics statement

The experimental protocols were approved by the Xiamen University Laboratory Animal Management Ethics Committee. All manipulations were strictly conducted in compliance with animal ethics guidelines and approved protocols.

### Strains and vector construction

N-terminally truncated HPV39 (P24838), HPV68 (AAZ39498.1) and HPV70 (P50793) L1 genes were cloned into the pTO-T7 expression vector by Gibson assembly, and plasmids were transferred into the ER2566 *E. coli* strain for L1 protein expression.

### Protein purification and particle assembly

ER2566 *E. coli* strains containing HPV39, −68, −70 WT or the chimeric L1 plasmid were cultured in LB liquid medium at 37 °C until reaching an OD_600_ of 0.6. Protein expression was then induced by the addition of isopropyl-β-D-thiogalactoside (IPTG, final concentration of 5 μM) and samples were further cultured at 24 °C for 10 h. Cells were then harvested by centrifugation (7000 × *g*, 10 min), resuspended in lysis buffer (50 mM Tris-Base [pH 7.2], 10 mM EDTA and 0.3 M NaCl) and lysed by ultrasonication. The ultrasonication output power was set at 45% of 500 W, and the ultrasonication time was 15 min per gram of *E. coli*, with a frequency of 2 s pulsed and 4 s paused. HPV L1 proteins were released from the cells and treated with 20 mM DTT for 12 h at room temperature. Then the supernatant was purified by chromatography using SP or XS sepharose (GE Healthcare). For the preparation of VLPs, the purified L1 protein was then dialyzed into a neutral buffer (10 mM pB6.5, 0.5 M NaCl) without DTT to allow VLP self-assembly and purified by Superdex 200.

### Sequence alignment and phylogenic tree construction

HPV39 (GenBank: P24838), HPV68 (AAZ39498.1), HPV70 (P50793), HPV6 (7EW5_A), HPV30 (YP_009508159.1), HPV34 (NP_041812.1), HPV51 (ACV88631.1), HPV58 (P26535.1), HPV66 (ABO76865.1), HPV67 (ALT54969.1), HPV69 (AHV83654.1), HPV73 (ALJ32570.1), HPV82 (ALJ32344.1), HPV85 (YP_009362313.1), HPV97 (ABO27083.1), and HPV11, −16, −18, −26, −31, −33, −35, −45, −52, −53, −56, −59^51^ L1 genes were from NCBI. L1 protein sequence alignment was performed using BLAST, and evolutionary tree building was performed by the Neighbor-joining method in MEGA X software (https://www.megasoftware.net).

### SDS-PAGE and western blotting

SDS-PAGE samples were diluted with an equal volume of sample buffer (125 mM Tris-HCl, pH 6.8, 4% (w/v) SDS, 20% (w/v) glycerol, 200 mM dithiothreitol, and 0.002% (w/v) bromophenol blue) to a final protein concentration of 0.3–0.5 mg/mL, heated at 80 °C for 10 min, and then loaded into the wells of a 10% separating gel. The gels were subjected to standard laboratory methods for gel electrophoresis (running at voltage of 80 V at concentration phase for 15 min, and then increase to 120 V at separation phase for additional 60 min in a BioRad MINI-PROTEAN Tetra system [BioRad Laboratories, CA, USA]) and stained with Coomassie Brilliant Blue R-250 (Bio-Rad) for 30 min at room temperature.

For western blotting, gels were transferred to nitrocellulose membranes, blocked with 5% skimmed milk for 1 h, and then incubated with HPV broad-spectrum linear monoclonal antibody 4B3 (1:1000 dilution) for 1 h at room temperature. Membranes were washed, and then incubated with goat anti-mouse alkaline phosphatase-conjugated antibodies (Abcam; Cambridge, UK; 1:5000 dilution) for 1 h. NBT/BCIP (Pierce Biotechnology; Rockford, IL) reagent was used to develop the color (5 min). All blots or gels derive from the same experiment and that they were processed in parallel. And source data are provided in Supplementary Fig. 4 and in the source data file.

### Transmission electron microscopy (TEM)

Samples were diluted to 0.15 mg/mL with HPV L1 storage buffer. Each sample (10 μL) was pipetted onto carbon-coated copper grids for 10 min, absorbing any residual liquid before adding dropwise 2% phosphotungstic acid (pH 6.4) and incubating the reaction for 5 min. An FEI Tecnai T12 TEM with an accelerating voltage of 120 kV was used to observe and measure HPV VLP morphology.

### High-performance size-exclusion chromatography (HPSEC)

The homogeneity of WT and chimeric VLPs was assessed using an Agilent 1200 high-performance liquid chromatography system, with a pre-installed TSK G5000 pwxl 7.5 mm × 300 mm column (TOSOH, Tokyo, Japan). The system flow rate was 0.5 mL/min, and the detection wavelength was set to 280 nm.

### Analytical ultra-centrifugation (AUC)

Sedimentation analysis was carried out on a Beckman XL-A analytical ultracentrifuge at 20 °C. 10 mM phosphate buffer pH 6.5 with 0.5 M NaCl was used as the reference solution. WT and chimeric VLPs were diluted to 0.5 mg/mL with reference solution, and centrifuged at 3951 × g using an An-60 Ti rotor (Beckman Coulter; Fullerton, CA). The sedimentation coefficient was determined using Sedfit software kindly provided by Dr. P. Schuck at the National Institutes of Health (Bethesda, MD).

### Differential scanning calorimetry (DSC)

The thermal stability of WT and chimeric VLPs was measured using MicroCal VP-Capillary differential scanning calorimetry (DSC) (GE Healthcare, MicroCal Products Group, Northampton, MA). Samples were diluted to 0.5 mg/mL with 10 mM phosphate buffer, pH 6.5, with 0.5 M NaCl. The temperature range was set as 10 °C to 90 °C, with a scanning rate of 100 °C/h. MicroCal Origin 8.0 software (Origin-Lab Corp., Northampton, MA) was used to analyze the DSC curve and the melting temperatures (Tm) of the samples.

### Indirect enzyme-linked immunosorbent assay (ELISA)

The wells of 96-well microplates were coated with WT or chimeric VLPs (300 ng per well) for 2 h at 37 °C and then blocked with 200 μL blocking solution for 2 h at 37 °C. The wells were then incubated with 100 μL of twofold serially diluted mAbs at a starting concentration of 1 μg/mL for 45 min at 37 °C. The wells were then washed and incubated with 100 μL horseradish peroxidase (HRP)-conjugated goat anti-mouse IgG antibody (diluted 1:5000 in HS-PBS, Abcam; Cambridge, UK), followed by 50 μL of 3, 3’, 5, 5’-tetramethylbenzidine liquid substrate (Sigma-Aldrich, St Louis, MO) per well for 10 min at 37 °C and the reaction was quenched with the addition of 50 µL 2 M H_2_SO_4_. An automated ELISA reader (TECAN, Männedorf, Switzerland) was used to detect the absorbance at 450 nm (reference, 620 nm). GraphPad Prism (GraphPad Software, San Diego, CA) were used to calculate the median effective concentration (EC_50_, ng/mL), which is the concentration of antibody that binds to 50% of the antigen.

### Cryo-electron microscopy (Cryo-EM) and three-dimensional (3D) reconstruction

The WT and chimeric VLPs (~2.0 mg/mL) were vitrified on Quantifoil holey carbon grids using an automated Thermo Fisher Vitrobot IV, and the cryo-EM images were collected using the Thermo Fisher TF30 electron microscope equipped with Thermo Fisher Falcon II and Falcon III cameras. The acceleration voltage was set to 300 kV and the magnification was set to 93,000×, resulting in a pixel size of 1.120 Å. Data were collected automatically by using Thermo Fisher EPU software. Icosahedral 3D reconstruction were performed by using AUTO3DEM^49^ or cisTEM^50^ software. The resolutions of the final 3D density maps were estimated based on the gold-standard FSC curve with a cut-off of 0.143^57^. Visualization of the density maps were performed with software ChimeraX^58^.

### Murine monoclonal antibodies (mAbs)

HPV WT and chimeric VLPs, formulated with Freund’s complete or incomplete adjuvant (20 μg/dose), were subcutaneously injected into BALB/c mice, with three injections delivered in 2-week intervals. The first injection used Freund’s incomplete adjuvant whereas the last two injections used Freund’s complete adjuvant. Cell fusion experiments were performed 2 weeks after a boost immunization. Fused hybridomas were isolated through hypoxanthine-aminopterin-thymidine medium (Sigma, Atlanta, GA) selection, and supernatants were screened by a indirect ELISA and PBNA for reactivity. Positive cells were cloned by limiting dilution at least three times until a single cell clone was attained.

The screened monoclonal hybridoma cells were injected into the peritoneal cavities of pristane-primed BALB/c mice. Ascitic fluid was collected after 9–12 days, and added with an equal volume of saturated ammonium sulfate solution, incubated at 4 °C for 30 min, centrifuged at 13,000 × *g* for 10 min, and the pellet was resuspended with 0.2 M Na_2_HPO_4_, and finally purified by protein A affinity chromatography. The resultant purified anti-HPV39 mAbs, anti-HPV68 mAbs and anti-HPV70 mAbs were diluted to 1.0 mg/mL in PBS and stored at −20 °C.

### HPV pseudoviruses preparation

The pvitro (for HPV 68 and 70) or pshell (for HPV39)-L1-L2 co-expression vector and the pcDNA3.1-EGFP expression vector used in the experiment were kindly provided by Dr. J. T. Schiller^59^. HPV39, -68 and -70 pseudoviruses (PsVs) were produced in HEK293FT cells, as follows. First, HEK293FT adherent cells were prepared with high activity and passaged one day before plasmid transfection, and when the cell density reached to 80–90%, then half of the medium was changed to ES serum-free dulbecco’s modified eagle medium (DMEM), the cell culture was incubated at 37 °C for 30 min. Second, the transfection system with 0.15 M NaCl buffer were prepared, 1 mL of the transfection system contains 10 μg of co-expressing plasmid carrying codon-optimized HPV L1-L2, 10 μg of pcDNA3.1-EGFP plasmid, and 65 μg of PEI (Yeasen Biotechnology (Shanghai) Co. Ltd.). The system was mixed and incubated at room temperature for 15 min, protected from light throughout. Third, the plasmid and PEI mixture were slowly added to the cells, 1 mL per plate, incubated at 37 °C for 4 h, then half of the medium was changed to ES serum-containing DMEM, and 0.5 mL ES serum was added to each cells plate, cultivated at 37 °C for 72 h. Fourth, the medium was aspirated and the cells were carefully washed with 2 mL DPBS, then resuspended with 4 mL DPBS and collected by centrifugation at 1,300 × g for 3 min at room temperature. Fifth, the cells were then lysed in 150 μL cell lysis buffer comprising 0.5% Brij58 (Sigma-Aldrich), 0.2% Benzonase (Merck Millipore; Darmstadt, Germany), 0.2% Plasmid-Safe ATP-Dependent DNase (Epicenter Biotechnologies, Madison, WI) and DPBS-Mg solution for 24 h at 37 °C. Finally, the above cell lysate was ice-bathed for 15 min, and 5 M NaCl of 0.19 times the volume of the lysate was added, ice-bathed for 15 min too after mixing, and then centrifuged at 1,300 × g for 10 min at 4 °C, the obtained supernatant was the pseudovirus used in neutralization assay.

### The measured of tissue culture infectious dose (TCID_50_)

The tissue culture infectious dose (TCID_50_) of the supernatant was then measured to determine the titers of the PsVs, calculated according to the classical Reed–Muench method^60^. First, HEK293FT cells in seeded into the wells of a 96-well plate at a density of 1.5 × 10^4^ cells/well and cultured for 4–6 h at 37 °C, and then a 10-fold gradient dilution was performed on the pseudovirus, with a total of 7 gradients. Second, the pseudovirus were added to the cells plate at the ratio of 100 μL/well, 8 wells of each gradient were repeated, cultured at 37 °C for 72 h. Third, the expression of EGFP in each well of each gradient were recorded by an inverted fluorescence microscope, and the proportional distance was calculated according to the Reed-Muench method: proportional distance = (infection rate at dilution next above 50%-50%) / (infection rate next above 50% - infection rate next below 50%); and the logarithm TCID_50_ can be obtained by multiplying the logarithm of the dilution coefficient the proportional distance, and plus the highest dilution of the virus with an infection rate higher than 50%. So the TCID_50_ value can be obtained too, and PsVs were diluted to 3 ×10^5^ TCID_50_/μL for PBNA assay.

### Pseudovirus-based neutralization assay (PBNA)

HEK293FT adherent cells were prepared with high activity and passaged one day before PBNA assay, and when the cell density reached to 80–90%, seeded into the wells of a 96-well plate at a density of 1.5 × 10^4^ cells/well and cultured for 4–6 h at 37 °C. Antibody sera were first diluted according to different dilution (2.5–1000) with ES serum-containing DMEM, and then subjected to 2-fold serial dilutions, for a total of 12 gradients, and PsVs were diluted with ES serum-containing DMEM to 3 × 10^5^ TCID_50_/μL. Sera and PsVs were then mixed at a ratio of 1:1 (60 μL each), and incubated at 37 °C for 1 h. Equal volumes (60 μL) PsV diluent and culture medium mixture was used as the negative control. Cells were then incubated with 100 μL of PsV-serum mixture for 72 h at 37 °C. The number of green fluorescent spots in each cell well was read with Elispot, and the PsV infection of the cell wells with the spots number lower than half of the average value of the sum of the negative control wells were considered to inhibited by serum neutralizing antibodies. Then the neutralization titers of the antibodies were calculated as the log10 of the highest sera dilution with a percentile of infection inhibition higher than 50%.

### Animals, immunizations and serological analysis

To initially assess the immunogenicity of chimeric VLPs, special pathogen-free (SPF) BALB/c female mice (*n* = 5) were immunized intraperitoneally three times at an interval of 2 weeks (week 0, 2 and 4) with WT or chimeric VLPs diluted in aluminum adjuvant prepared at three different doses (5, 1 or 0.2 μg per dose). In the mixed WT VLP group, the dose of each VLP was equal to that of the same VLP in the single-type groups. Serum samples were collected at week 8, and the neutralizing titers were analyzed by PBNA.

For ED_50_ analysis, SPF BALB/c female mice (*n* = 8) were vaccinated via an intraperitoneal injection of a single dose (0.300, 0.100, 0.033 or 0.011 μg) of WT or chimeric VLPs diluted in aluminum adjuvant. In the mixed WT VLP group, the dose of each VLP was equal to that of the same VLP in the single-type groups. Serum samples were taken 5 weeks after immunization, and the ED_50_, (i.e., the immune dose that causes 50% seroconversion in the mice), were analyzed based on a dose-response curve using the Reed–Muench model^60^.

### Statistical methods

SPSS statistics (International Business Machines Corporation, New York, USA) was used for statistical analysis of data sets. All data were analyzed by one-way analysis of variance (ANOVA) and are presented as the mean ± standard deviation (SD). *P* values < 0.05 was considered statistically significant.

### Reporting summary

Further information on research design is available in the Nature Research Reporting Summary linked to this article.

## Supplementary information

NPJVACCINES-01815R2_Supplementary information_all_clean__Oct-10th

Dataset 1

Reporting Summary

## Data Availability

All data supporting the findings of this study are available from the corresponding author on reasonable request. The cryo-EM density maps for HPV39, HPV68, HPV70, H39–68FG-70DE and H39–68FG-70HI have been deposited in the Electron Microscopy Data Bank (EMDB) with accession codes of EMDB-34239, EMDB-34240, EMDB-34241, EMDB-34242 and EMDB-34243, respectively. A reporting summary for this article is available as a Supplementary Information file. Source data are also provided with this paper.
