# Supplementary material for: Characterization of an Escherichia coli-derived triple-type chimeric vaccine against human papillomavirus types 39, 68 and 70
Source: NPJ Vaccines. 2022 Oct 31;7:134. doi: 10.1038/s41541-022-00557-y (PMC9622684; doi:10.1038/s41541-022-00557-y)
Supplement: Supplementary file 1 — NPJVACCINES-01815R2_Supplementary information_all_clean__Oct-10th [file 41541_2022_557_MOESM1_ESM.pdf]

Supplementary information

Characterization of an *Escherichia coli*-derived triple-type chimeric vaccine against human papillomavirus types 39, 68 and 70

Ciying Qian[1,2,#], Yurou Yang[1,2,#], Qin Xu[1,2,#], Zhiping Wang[1,2], Jie Chen[1,2], Xin Chi[1,2], Miao Yu[1,2], Fei Gao[1,2], Yujie Xu[1,2], Yihan Lu[1,2], Hui Sun[1,2], Jingjia Shen[1,2], Daning Wang[1,2], Lizhi Zhou[1,2], Tingting Li[1,2], Yingbin Wang[1,2], Qingbing Zheng[1,2], Hai Yu[1,2], Jun Zhang[1,2], Ying Gu[1,2,*], Ningshao Xia[1,2,*], Shaowei Li[1,2,*]

[*] To whom correspondence should be addressed. Email: shaowei@xmu.edu.cn (S.L.) or nsxia@xmu.edu.cn (N.X.) or guying@xmu.edu.cn (Y.G.)

This PDF file includes:

Supplementary Figures 1 to 4
Supplementary Tables 1 to 4

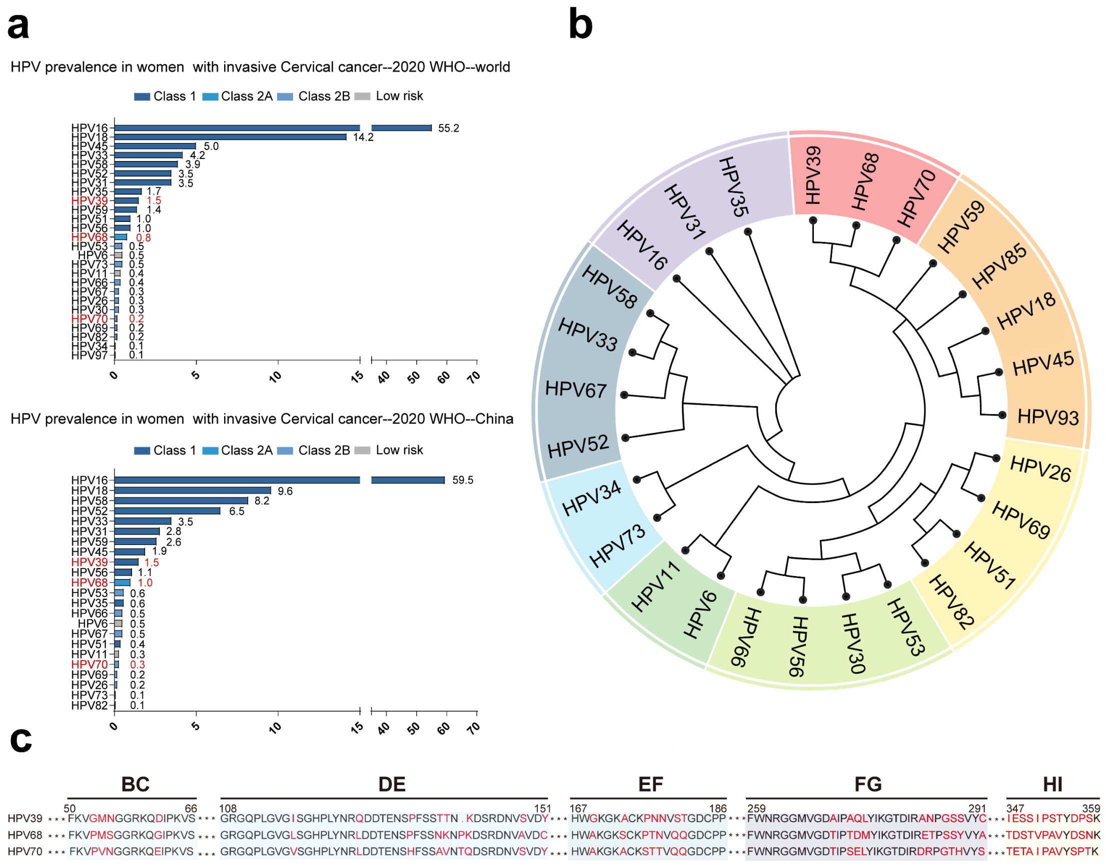

Supplementary Figure 1. Evolution analysis of 27 HPV L1 proteins and the prevalence of these types among women with invasive cervical cancer. **a** The proportion of various HPV types in women with invasive cervical cancer in China and the world according to the 2021 WHO report (URL: https://hpvcentre.net/statistics/reports/XWX.pdf?t=1646934138325). HPV types are divided into classes according to carcinogenicity: class 1 (carcinogenic), class 2A (probably carcinogenic), class 2B (possibly carcinogenic) and class 3 (low-risk). **b** Phylogenetic tree of 27 HPV types based on the HPV L1 amino acid sequence. According to the closeness of the relationship, 25 types of high-risk HPV and 2 types of low-risk HPV can be divided into 8 evolutionary clusters. **c** Comparison of the amino acid sequences of the surface loops (BC, DE, EF, FG and HI-loop) of HPV39, -68 and -70. A red font is used to indicate that the three amino acids are different, whereas a black font indicates that they are the same.

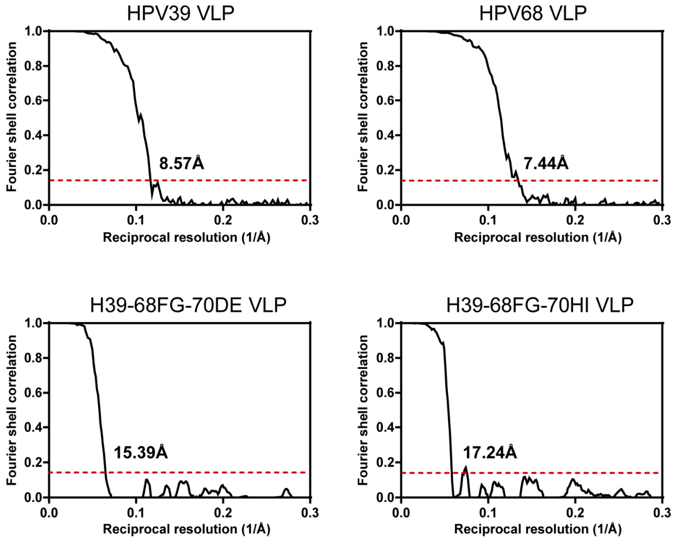

Supplementary Figure 2. Gold standard FSC curves of the icosahedral reconstructions of HPV39, HPV68, HPV70, H39-68FG-70DE and H39-68FG-70HI at resolutions of 8.57Å, 7.44 Å, 10.67 Å, 15.39 Å, and 17.24 Å, respectively, with a cut-off threshold of 0.143.

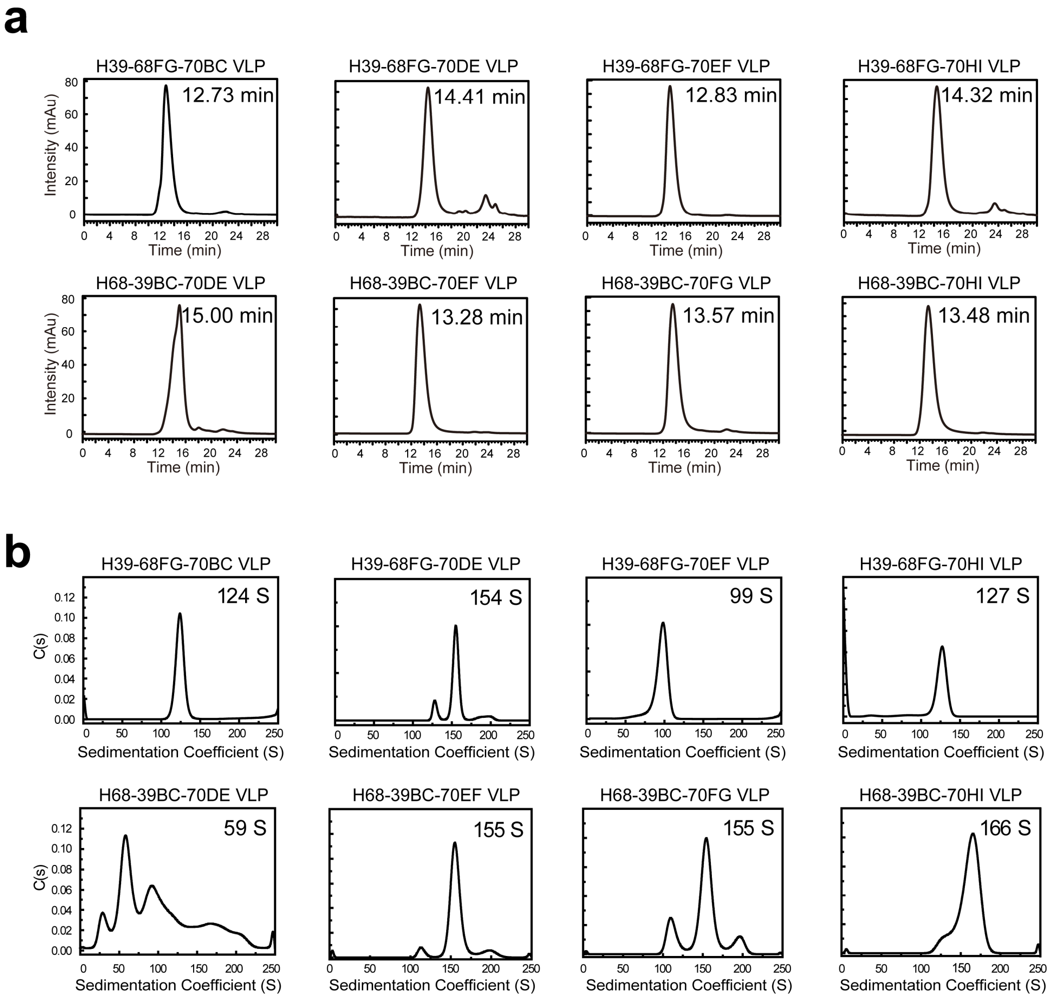

Supplementary Figure 3. Characterization of HPV39/68/70 chimeric VLPs. **a-b** High-performance size-exclusion chromatography (HPSEC) profiles (**a**) and analytical ultracentrifugation sedimentation (AUC) profiles (**b**) of the chimeric VLPs.

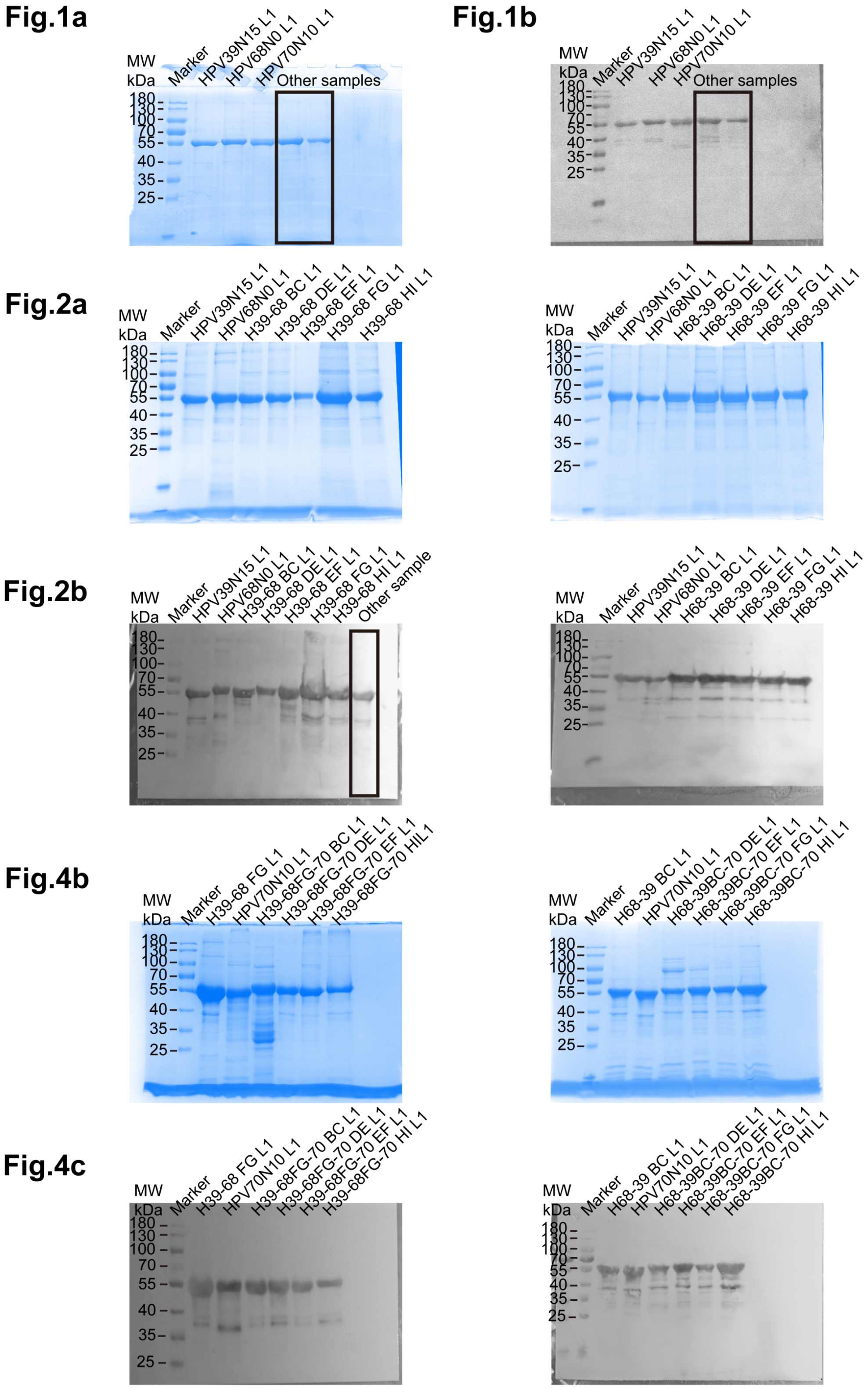

Supplementary Figure 4. Uncropped original scans of blots and gels from Figure 1a, 1b, 2a, 2b, 4b and 4c.

Supplementary Table 1. Statistics of cryo-EM data collection and refinement.

	HPV39	HPV68	HPV70	H39-68FG-70DE	H39-68FG-70HI
Microscope	TF30	TF30	TF30	TF30	TF30
Camera	Falcon III	Falcon III	Falcon III	Falcon III	Falcon III
Magnification	93,000	93,000	93,000	93,000	93,000
Voltage (kV)	300	300	300	300	300
Electron exposure (e/A^2)	30	30	30	30	30
Defocus range (μm)	-1.1~-4.1	-0.9~-4.4	-0.5~-2.7	-0.5~-4.7	-1.6~-5.2
Pixel size (Å)	1.120 Å	1.120 Å	1.120 Å	1.120 Å	1.120 Å
Micrographs	272	512	577	672	303
Final particle (no.)	7,651	7,456	2,051	6,410	1,933
Symmetry imposed	I2	I2	I2	I2	I2
Map resolution (Å)	8.57	7.44	10.67	15.39	17.24
FSC threshold	0.143	0.143	0.143	0.143	0.143
Map sharpening B factor ($Å^2$)	-200	-250	0	0	0

Supplementary Table 2. Half-Effective Dose (ED_{50}) in Mice for Aluminum Adjuvant Containing Chimeric or WT VLPs of HPV39 and HPV68

Antigen	Dose	PsV39		PsV68	
(VLP)	(μg)	Seroconversion no./ inoculated no.	ED_{50} (μg)	Seroconversion no./ inoculated no.	ED_{50} (μg)
H39-68FG	0.900	6/8	0.091	3/8	0.405
	0.300	6/8		4/8	
	0.100	6/8		2/8	
	0.033	1/8		1/8	
H68-39BC	0.900	4/8	0.395	8/8	0.021
	0.300	4/8		8/8	
	0.100	1/8		8/8	
	0.033	1/8		7/8	
H68-39HI	0.900	0/8	＞0.900	7/8	0.028
	0.300	0/8		8/8	
	0.100	0/8		7/8	
	0.033	0/8		6/8	
HPV39	0.900	8/8	0.022	0/0	＞0.900
	0.300	8/8		0/0	
	0.100	6/8		0/0	
	0.033	8/8		0/0	
HPV68	0.900	0/0	＞0.900	8/8	0.023
	0.300	0/0		8/8	
	0.100	0/0		7/8	
	0.033	0/0		7/8	
HPV39&68	0.900	7/8	0.021	7/8	0.023
	0.300	8/8		8/8	
	0.100	8/8		8/8	
	0.033	8/8		7/8	

Supplementary Table 3. Half-Effective Dose (ED_{50}) in Mice for Aluminum Adjuvant Containing Chimeric or WT VLPs of HPV39, HPV68 and HPV70

Antigen (VLP)	Dose (μg)	PsV39 Seroconversion no./ inoculated no.	PsV68 ED_{50} (μg)	Seroconversion no./ inoculated no.	PsV70 ED_{50} (μg)	Seroconversion no./ inoculated no.	ED_{50} (μg)
H39-68FG-70DE	0.900	8/8	0.021	6/8	0.030	7/8	0.040
	0.300	7/8		8/8		7/8	
	0.100	8/8		7/8		5/8	
	0.033	8/8		6/8		6/8	
H39-68FG-70EF	0.900	3/8	0.900	7/8	0.024	4/8	0.611
	0.300	1/8		7/8		3/8	
	0.100	0/8		7/8		0/8	
	0.033	1/8		8/8		0/8	
H39-68FG-70HI	0.900	7/8	0.022	8/8	0.148	7/8	0.047
	0.300	7/8		8/8		6/8	
	0.100	8/8		1/8		8/8	
	0.033	8/8		1/8		4/8	
HPV39	0.900	8/8	0.019	0/8	> 0.900	0/8	> 0.900
	0.300	8/8		0/8		1/8	
	0.100	8/8		0/8		0/8	
	0.033	8/8		1/8		0/8	
HPV68	0.900	0/8	> 0.900	8/8	0.021	2/8	0.900
	0.300	0/8		8/8		2/8	
	0.100	1/8		8/8		0/8	
	0.033	1/8		7/8		2/8	
HPV70	0.900	2/8	> 0.900	0/8	> 0.900	8/8	0.019
	0.300	0/8		0/8		8/8	
	0.100	1/8		0/8		8/8	

	0.033	1/8		0/8		8/8	
HPV39&68&70	0.900	8/8	0.021	8/8	0.021	8/8	0.021
	0.300	8/8		8/8		8/8	
	0.100	7/8		7/8		7/8	
	0.033	8/8		8/8		8/8	

Supplementary Table 4. Characterization of HPV39, HPV68 and HPV70 mAb panel and antigenicity assay of HPV39/68/70 chimeras.

Immunogen for antibody generation (VLP)	mAbs	Isotype	mAb reactivity of WT HPV39, HPV68, HPV70 and HPV39/68/70 chimeras (EC_{50}, ng)				
			HPV39	HPV68	HPV70	HPV39-68FG-70DE	HPV39-68FG-70HI
HPV39	2D11	IgG1	30.1	>10000	>10000	19595.0	>10000
	3A2	IgG2b	2109.0	>10000	>10000	>10000	>10000
	3C12	IgG1	51.4	>10000	>10000	>10000	>10000
	5F10	IgG1	20.4	>10000	>10000	>10000	>10000
	19B8	IgG2b	6.3	>10000	>10000	4.3	6.4
	20F8	IgG2a	490.1	1026.0	>10000	1005.0	690.5
HPV68	5E10	IgG1	>10000	4.4	>10000	>10000	192.2
	3B9	IgG1	>10000	1.6	>10000	>10000	289.6
	9F4	IgG2a	>10000	20.2	>10000	>10000	63.0
	15E2	IgG1	>10000	2.4	>10000	8915.0	6877.0
	1C1	IgG1	>10000	6.5	>10000	434.0	>10000
	13F9	IgG1	>10000	12.5	>10000	331.6	1782.0
	15E9	IgG1	>10000	18.6	>10000	4216.0	>10000
HPV70	2A9	IgG1	>10000	>10000	5.0	20.4	2597.00
	16A3	IgG1	>10000	>10000	7.8	38.9	3443.00
	16D11	IgG1	>10000	>10000	6.8	38.3	3722.00
	12B11	IgG1	>10000	>10000	2.0	>10000	1.3
	16C4	IgG1	>10000	>10000	4.0	18.7	>10000
	10B1	IgG2b	>10000	>10000	2.6	12.6	2362.0
	10C3	IgG2b	>10000	>10000	17.2	3722.0	576.1
	10C10	IgG1	>10000	>10000	65.2	3553.0	2833.0
	7B7	IgG2b	>10000	>10000	1.6	9292.0	3053.0
	8C10	IgG1	>10000	>10000	2.0	4152.0	2761.0
